# *Curcumin* ameliorates peritoneal fibrosis via inhibition of transforming growth factor-activated kinase 1 (TAK1) pathway in a rat model of peritoneal dialysis

**DOI:** 10.1186/s12906-019-2702-6

**Published:** 2019-10-23

**Authors:** Jun-Li Zhao, Ting Zhang, Xia Shao, Jun-Jun Zhu, Mei-Zi Guo

**Affiliations:** 1 Department of Nephrology, Shanghai University of Medicine & Health Sciences Affiliated Zhoupu Hospital, Pudong New District, Shanghai, 201318 China; 2 Department of Geriatrics, Shanghai University of Medicine & Health Sciences Affiliated Zhoupu Hospital, Pudong New District, Shanghai, 201318 China

**Keywords:** Peritoneal dialysis (PD), Peritoneal fibrosis (PF), Curcumin, Transforming growth factor-activated kinase-1 (TAK1), Epithelial-mesenchymal transition (EMT)

## Abstract

**Background:**

Peritoneal fibrosis (PF) remains a serious complication of long-term peritoneal dialysis (PD). The goal of this study was to investigate the anti-fibrotic effects of curcumin on the PF response to PD and its’ mechanism.

**Methods:**

Male Sprague–Dawley rats were infused with 20 mL of 4.25% glucose-based standard PD fluid for 8 consecutive weeks to establish PF model and then divided into five groups: Control, received sham operation and 0.9% physiological saline; PD, received 4.25% standard PD fluid; Curcumin, PD rats injected intraperitoeally with curcumin for 8 weeks at doses of 10, 20 or 40 mg/kg. Masson’s staining was performed to evaluate the extent of PF. Peritoneal Equilibration Test (PET) was conducted to assess ultrafiltration volume (UFV) and mass transfer of glucose (MTG), quantitative RT-PCR, and immunohistochemistry or western blotting were performed to measure the expression levels of inflammation and fibrosis-associated factors. We also detected the TGF-β1 in peritoneal fluid by ELISA.

**Results:**

Compared with the control group, the PD rats showed decreased UFV (2.54 ± 0.48 to 9.87 ± 0.78 mL, *p* < 0.05] and increased MTG (18.99 ± 0.86 to 10.85 ± 0.65 mmol/kg, *p* < 0.05) as well as obvious fibroproliferative response, with markedly increased peritoneal thickness (178.33 ± 4.42 to 25.26 ± 0.32um, *p* < 0.05) and higher expression of a-SMA, collagen I and TGF-β1. Treatment with curcumin significantly increased UFV, reduced MTG and peritoneal thickness of PD rats. The elevated TGF-β1 in peritoneal fluid of PD rats was significantly decreased by curcumin. It attenuated the increase in protein and mRNA of TGF-β1, α-SMA and collagen I in peritoneum of PD rats. The mRNA expressions of TAK1, JNK and p38, as well as the protein expressions of p-TAK1, p-JNK and p-p38 in peritoneum of PD rats were reduced by curcumin.

**Conclusions:**

Present results demonstrate that curcumin showed a protective effect on PD-related PF and suggest an implication of TAK1, p38 and JNK pathway in mediating the benefical effects of curcumin.

## Background

Peritoneal dialysis (PD) is currently recognized as one of the effective methods of renal replacement therapy for patients suffering from end-stage renal disease (ESRD). However, long-term exposure of the peritoneal membrane to nonbiocompatible peritoneal dialysis fluid, including high concentrations of glucose and lactate, glucose degradation products (GDPs), and low pH, can result in functional and structural abnormalities of peritoneum, eventually leading to the PD failure [1]. High glucose can lead to inflammation and peritoneal fibrosis (PF) [2], increase the expression of transforming growth factor-β1 (TGF-β1) [3], and stimulate a complex process of peritoneal epithelial-mesenchymal transition (EMT) in vivo [4]. EMT of mesothelial cells is an reversible process in which epithelial cells transdifferentiate into cells with mesenchymal characteristics, is widely considered to be a crucial process in fibrosis [5, 6].

Currently, no appropriate methods to block peritoneal fibrosis have been approved in clinical practice. Most studies so far have focused on Chinese medicine materials as an alternative treatment which shows to suppress the pro-inflammatory and pro-fibrotic pathway and control PF in several in vivo and/or in vitro studies [7, 8]. Curcumin, a polyphenol isolated from the *Curcuma longa* plant, is commonly known as turmeric in Asia. Curcumin is one of active ingredients in turmeric, with many pharmacological functions on tumor, inflammation, and oxidative stress [9]. Curcumin demonstrates wide suppressive effects on fibrosis through reducing the production of TGF-β1, such as pulmonary fibrosis [10], liver fibrosis [11], and oral submucous fibrosis [12]. Recent studies have found that curcumin shows anti-fibrotic effects on renal fibrosis through interfere with TGF-β/Smad signaling pathways, preventing inflammation initiation, inhibiting EMT, and resolving ECM excess deposition in animal models [13]. In addition, there is no toxicity concern rising when curcumin is taken at the recommended doses, which increased the potential of therapeutic agent of this compound. However, the protective effects and exact molecular mechanisms of curcumin on peritoneal fibrosis induced by peritoneal dialysis still need to be elucidated. The Smad signaling pathway is widely accepted as a canonical pathway induced by TGF-β1 in the induction of fibrosis. The canonical Smad pathway involves activation of Smad2–3 through recruitment and phosphorylation by activated TβRI. The recruitment of Smad2–3 to the receptor complex is mediated by auxiliary proteins, such as Smad anchor for receptor activation (SARA). Smad2–3 is subsequently released from the receptor complex to interact with Smad4 to transmit TGF-β1 signals [14]. Actually, the balance between TGF-β1 activated Smad2–3 and BMP-activated Smad1–5–8 controls the peritoneal EMT and fibrosis status [15]. Besides this, a large body of evidence has demonstrated that various Smad-independent signaling pathways are involved in the development of EMT and fibrosis [16]. Transforming growth factor-activated kinase-1 (TAK1), a serine/threonine kinase, emerged as a critical upstream signaling molecule in TGF-β-induced Smad-independent signaling pathways [17]. A recent study by Strippoli [18] showed that TAK1 as a main biochemical mediator mediated EMT and fibrosis in mesothelial cells from human peritoneum.

These findings suggest that TGF-β1/TAK1 signaling pathway may involved in suppression of PF by curcumin. To test this hypothesis, we first determined the effects of curcumin on PF and function in PD model rats. Second, TGF-β1 and TAK1 was examined in the peritoneal fluid and peritoneum of rats. Third, the expression of pTAK1 and downstream proteins p-JNK and p-p38 were determined in PD rats with curcumin treatment.

## Methods

### Reagents and antibodies

Curcumin (No. C7727, purity > 99%) was purchased from Sigma Chemical Corp (St. Louis, MO, USA), dissolved at a concentration of 100 mg/mL in DMSO, and stored at − 20 °C. Before use, the curcumin solution was dissolved in a physiological saline solution at a concentration of 25 mg/mL, and then diluted with peritoneal solution for further intraperitoneal injection. Peritoneal dialysis fluid (Dianeal PD-2 peritoneal dialysis solution with 4.25% dextrose, pH 5.2) was purchased from Baxter Medical Co., Ltd. (Guangzhou, China). Rat TGF-β1 and ELISA kit was purchased from R&D Systems (Minneapolis, MN, USA). Anti-rat TGF-β1, α-SMA and collagen I antibodies were purchased from Sigma Chemical Co. (St. Louis, MO, USA). Primary antibodies to p-TAK1, p-JNK and p-p38 were purchased from Santa Cruz (Santa Cruz, USA).

### Animals

Male Sprague–Dawley rats (200–250 g body weight, 8-week-old) were purchased from Shanghai SLRC Laboratory Animal Co., Ltd. (Shanghai, China). Rats were housed in polycarbonate cages maintained at 24 °C, and were given free access to water and diet, with 40–70% humidity and 12 h/12 h light/dark cycles. All animal experiments conformed to the British Home Office Regulations (Animal Scientific Procedures Act 1986) for the care and use of animals. The experimental procedures were approved by animal ethical committee of Shanghai University of Medicine & Health Sciences affiliated Zhoupu Hospital.

### Peritoneal dialysis model

Peritoneal dialysis model was established as described previously, with minor modifications. Briefly, a self-made catheter for PD (medical intravenous tubing with a heparin lock on one side and with many side holes on the other) was inserted 2 cm below the costarum. The end with holes was inserted into the abdominal cavity of rats. A subcutaneous tunnel was formed in unison with incision to the midpoint between the two ears of rats. Normal saline solution (20 mL) was administered via the catheter to check for any possibility of leakage. The catheter implantation was established successfully if the previously administered saline solution flowed out in a smooth manner. After 1 week, the rats were daily infused with 4.25% glucose dialysis solution (100 mL/kg) for 8 weeks, with intraperitoneal injection of LPS (0.6 mg/kg) at 1, 3, 5 and 7 days. After instillation of dialysis solution, the catheter was heparinized with daily injection of 10 IU heparin and 5 mg cephazolin to avoid catheter blockage and infection.

A total of 50 rats were randomly divided into five groups, with ten rats in each group: Control group, which received sham operation and 0.9% physiological saline through inserted PD catheters for 8 weeks; PD group, which received daily infusion of 4.25% PD fluids (100 mL/kg) for 8 weeks; Curcumin group, which received daily infusion of 4.25% PD fluids (100 mL/kg) and intraperitoneal injection of Curcumin (10, 20, 40 mg/kg) for 7 weeks. Curcumin treatments were started on the 8st day after the first instillation of PD fluid. After 8 weeks of PD, the rat was sacrificed by spinal dislocation, the abdomen was opened by a midline incision, and the entire anterior abdominal wall was removed at the contralateral side to the tip of the implanted catheter. The whole tissue adjacent to the liver was fixed in 10% neutral-buffered formalin and embedded in paraffin for further examination.

### Peritoneal function assessment

Peritoneal Equilibration Test (PET) was performed to determine ultrafiltration volume (UFV) and mass transfer of glucose (MTG) in rats in each group. At the end of the experiments, each rat was infused 25 mL of 4.25% PD fluids, and 1 ml dialysate was reserved to detect UFV and MTG of the rats from the initial period at the beginning. A longitudinal incision was made in the abdominal wall following a 4-h period. Four hours later, Most of the fluid within the peritoneal cavity was extracted with a syringe and was subsequently measured. The rat was sacrificed, the peritoneum was opened, and the entire fluid content was dried by gauze, which was weighed immediately. UFV (UF, mL) = the drained volume (mL) + (the weight of wet gauze - the weight of dry gauze)- 25 mL. MTG (nmol/kg) = (glucose concentration at the beginning of PD × the initial volume of injected dialysate) – (glucose concentration after PD × the final volume of reserved dialysate). Meanwhile, the peritoneal fluid was collected with sterile syringe for further biochemistry testing.

### Enzyme-linked immunosorbent assay (ELISA)

ELSA was performed to determine the concentrations of TGF-β1 in peritoneal fluid after the last 4-h infusion of 4.25% PD by ELISA kit according to the manufacturer’s instructions. After the coloration, the absorbance (A) value at 450 nm wavelength was measured to establish a standard curve, and the actual TGF-β1 concentration were determined and expressed as pg/mL.

### Histology

Sections (2 μm) of the paraffin-embedded, fixed peritoneum sample tissues were deparaffinized, hydrated in ethyl alcohol, and washed in tap water. Masson’s trichrome staining were performed to quantify the pathological condition and peritoneum thickness. The mean peritoneal thickness from each rat was calculated from at least 6 sections. The image was analyzed using Image-Pro Plus Software (Media Cybernetics Inc., Bethesda, USA).

### Immunohistochemistry

The parietal peritoneum was fixed with 4% paraformaldehyde for 4 h, and embedded with paraffin. Then paraffin-embedded peritoneum tissue was serially sectioned (thickness 2 μm). After deparaffinization and rehydration, antigenic recovery was performed by heat and endogenous peroxidase activity was blocked by incubation with 3% H_2_O_2_. Sections were incubated with primary antibodies against TGF-β1, α-SMA and collagen I (1:100) overnight at 4 °C. In the second day, sections were incubated with biotinylated HRP conjugated secondary antibody (1:100) at room temperature for 2 h, and stained with diaminobenzidine (DAB) and hematoxylin. More than five vision fields were randomly selected in each section and photographed. Image-Pro Plus Software was used to calculate the positive cells of TGF-β1 to evaluate cell density (cells/mm^2^), and to calculate the staining area of α-SMA and collagen I, which was normalized to area of vision fields.

### Quantitative real-time PCR

The tissues of the abdominal wall were homogenized under liquid nitrogen and RNA was isolated using TRIzol reagent. cDNA was obtained by reverse transcription from RNA using the enzyme III (Life Technologies). Real-time PCR primers for TGF-β1, α-SMA, collagen I, TAK1, JNK, p38 and GAPDH were synthesized by Shanghai Invitrogen (Table 1). Reaction systems contained 2 × SYBR® Premix Ex TaqTM II (Tli RNaseH Plus) 10 μL, 50 × ROX Reference Dye 0.4 μL, template cDNA 2 μL, upstream and downstream primers 0.4 μL, and deionized water 7.2 μL. Amplification conditions were set as follows: 95 °C pre-denaturation for 30 s, followed by 40 cycles of 95 °C degeneration for 5 s and 60 °C annealing for 30 s. Human GAPDH gene was used as an internal reference. ΔΔCt = (target gene - internal reference) CT value - (control group target gene - control internal reference) CT value; relative mRNA expression amount = 2 - ΔΔCt × 100% (16).

**Table 1:** Primer sequences

Genes	Forward primer	Reverse primer
TGF-β1 (168 bp)	AGGGCTACCATGCCAACTTC	CCACGTAGTAGACGATGGGC
CTGF (100 bp)	TAGCTGCCTACCGACTGGAA	GTCTTAGAACAGGCGCTCCA
α-SMA (177 bp) TAK1 (190 bp)	GGAGCATCCGACCTTGCTAA CCATCCCAATGGCGTATCTTACA	CCATCTCCAGAGTCCAGCAC TCATCCTGGTCCAATTCTGCAA
JNK (161 bp)	CGGAACACCTTGTCCTGAAT	TCGCCTGACTGGCTTTAAGT
p38 (206 bp)	CCGAGCGATACCAGAAC	CACATCCAACAGACCAATC
GAPDH (253 bp)	ACAGCAACAGGGTGGTGGAC	TTTGAGGGTGCAGCGAACTT

### Western blotting

The peritoneal tissues of rats were lysed in RIPA buffer with protease inhibitors. Then centrifugation was performed at 12000×g and 4 °C for 10 min, and the supernatants were collected to quantify total protein concentration using a total protein assays kit (Pierce, Rockford, IL, USA). Proteins (50 μg) were separated by 12% SDS-PAGE gel and then transferred to a PVDF membrane (Millipore, Bedford, USA), which was blocked with 5% skim milk in TBST at 4 °C. Then membrane was incubated with primary antibodies against p-TAK1, p-JNK and p-p38 (all 1:1000 dilutions) at 4 °C overnight. In the second day, the membrane was then incubated with HRP-labeled secondary antibody (IgG) (1:1000 dilution) for 1 h at room temperature. Band densities were visualized using a chemiluminescent detection system (ECL, Amersham Life Sciences, Buckinghamshire, UK) and Bio-Rad chemidoc XRS (Bio-Rad, USA). The gray value of target protein bands were normalized to that of GAPDH.

### Statistical analysis

Data are expressed as means ± standard deviation from at least three independent experiments, and analyzed by SPSS 19 statistical software. Differences between treatment groups were analyzed by t-test or analysis of variance (ANOVA), followed by Tukey’s post hoc test. Two-tailed *P* value < 0.05 was considered as statistical significance.

## Results

### Effect of Curcumin on peritoneal fibrosis in PD rats

**Fig. 1:**
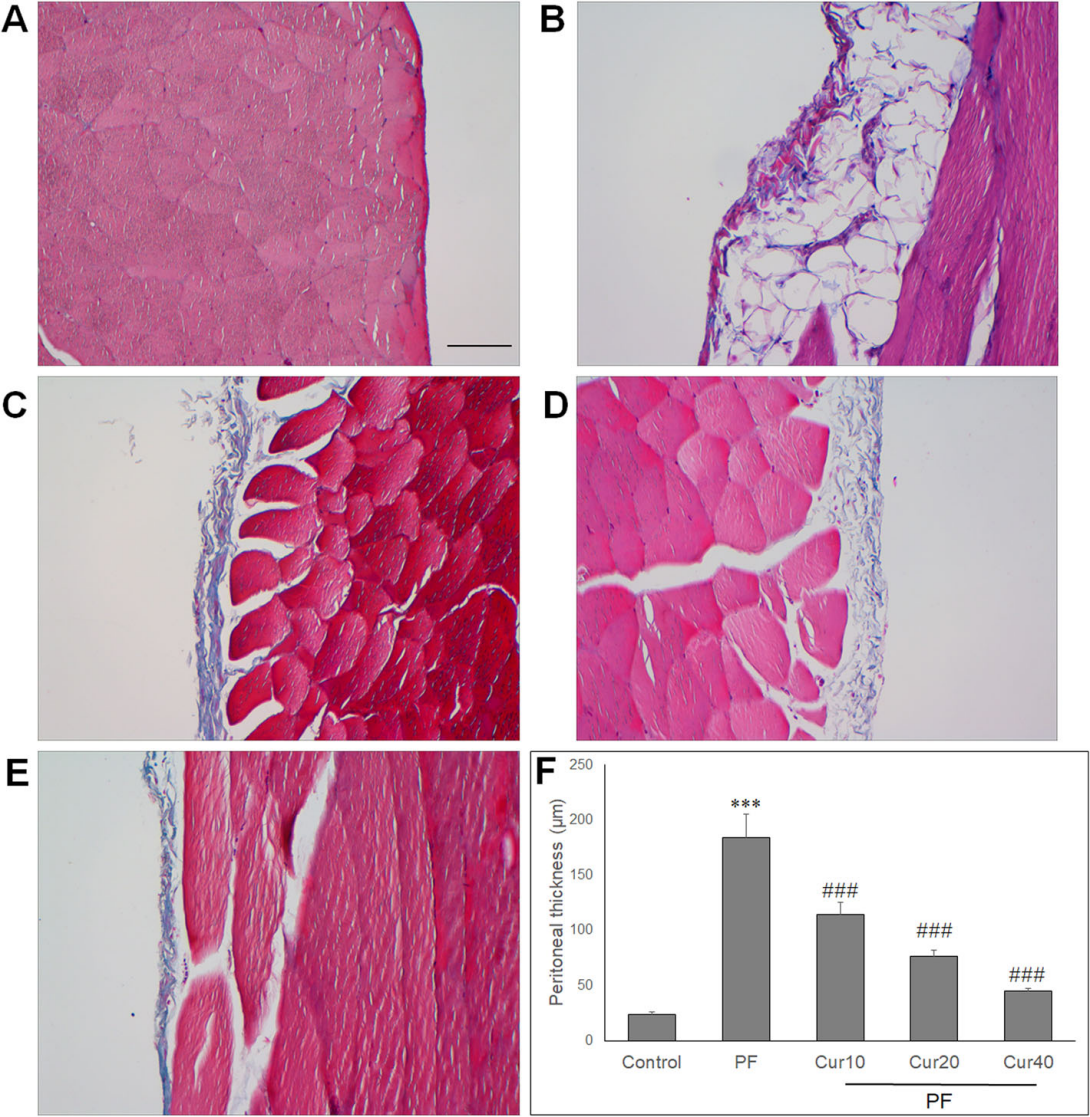
Effects of Curcumin on the peritoneal thickness of PF rats. Peritoneum was stained with Masson’s trichrome to measure the thickness of submesothelial zone. Representative photomicrographs of peritoneal samples was shown. **a** Control rats showed a thin submesothelial layer of peritoneum, without any morphological changes. **b** PF rats showed a marked thickening of the submesothelial compact zone. Curcumin prevented the thickening of submesothelial zone with low (**c**), moderate (**d**) and high (**e**) doses. **d** Quantification analysis of the effect of curcumin on peritoneal thickness. Bar, 100 μm. Magnification, 200×. Data are represented as means ± SD for 12 rat of each group. ANOVA was performed and followed with Tukey’s post hoc test. ***P < 0.001 vs. control group; ###*P* < 0.001 vs. PF group

The peritoneum pathology was evaluated by Masson’s Trichrome staining after 8 weeks of PD in rats. In the peritoneum of control rats, there were a monolayer of mesothelial cells and a thin layer of connective tissue underneath (Fig. 1a). Otherwise, in the peritoneal sample of PD rats, there were extensive interstitial fibrosis, mesothelial denudation and markedly thickened compact zone (Fig. 1b). However, curcumin treatment reduced the peritoneal thickening of PD rats at different doses (Fig. 1c, d, e). Quantification analysis showed significant thickening of peritoneum in PD rats compared with control rats, and curcumin significantly reduced peritoneal thickness in PD rats (*P* < 0.05) (Fig. 1f).

### Curcumin preserved peritoneal transport function

**Fig. 2:**
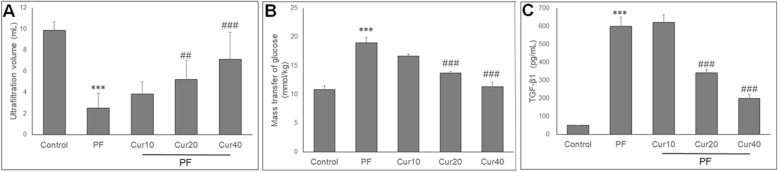
Effects of Curcumin on peritoneum function and TGF-β1 content in peritoneal effluence. Curcumin attenuates the decrease in ultrafiltration volume and (**a**) increase in mass transfer of glucose (MTG) (**b**) in PF rats. ELISA showed that compared with control group, PF rats showed significantly higher expression of TGF-β1 (**c**) in peritoneal effluence, and these changes can be attenuated by curcumin. ANOVA was performed and followed with Tukey’s post hoc test. ***P < 0.001 vs. control group; ##*P* < 0.01, ###*P* < 0.001 vs. PF group

To evaluate whether curcumin had an impact on preserving peritoneal function, UFV and MTG of rats were analyzed. PD rats showed impaired peritoneal function, as demonstrated by significantly lower UFV and higher MTG than control groups (*P* < 0.05) (Fig. 2a, b). *Curcumin* treatment preserved peritoneal function, with significantly increased UFV and reduced MTG level at moderate (20 mg/kg) and high (40 mg/kg) doses compared with PD rats (*P* < 0.05). Meanwhile, this effect displayed in a dose-dependent manner.

### Effect of Curcumin on TGF-β1 expression in PD rats

**Fig. 3:**
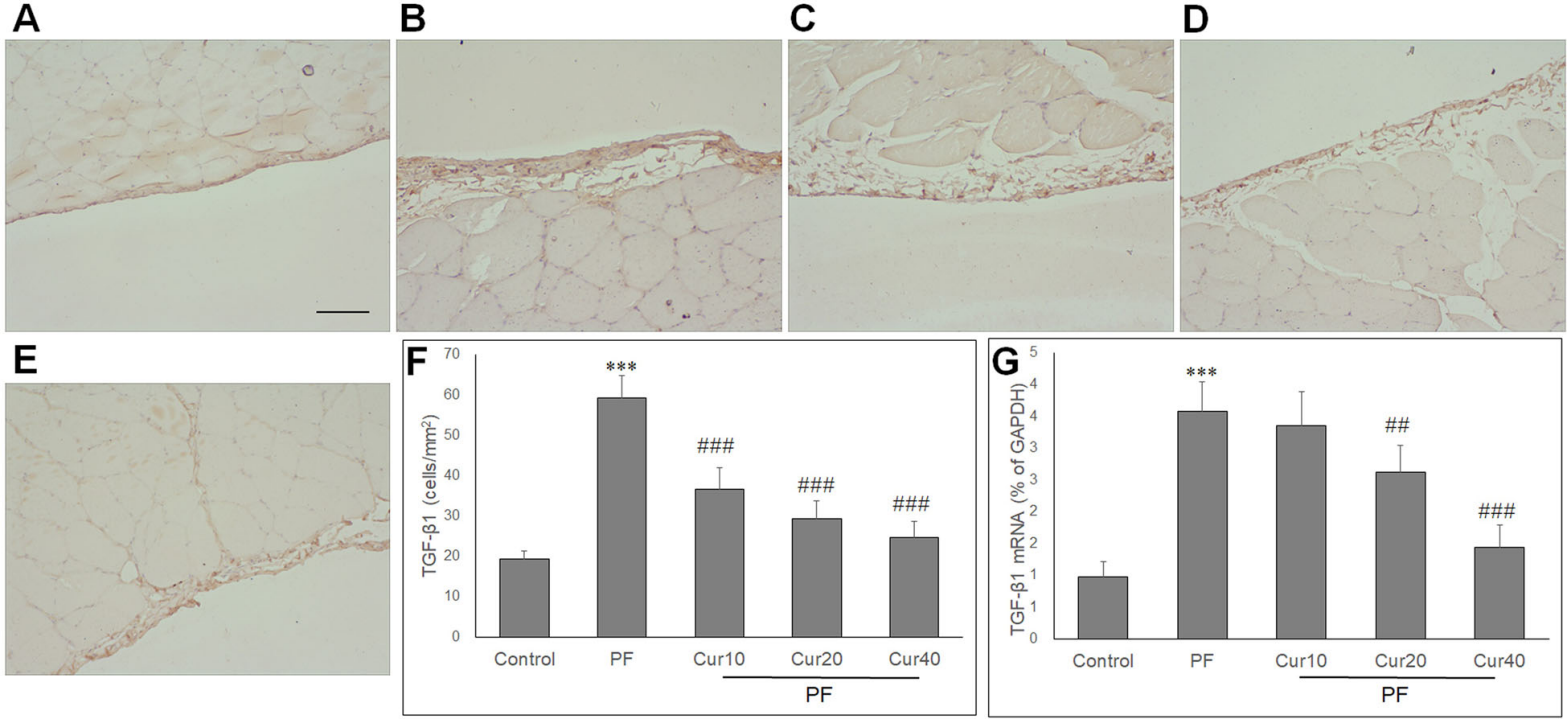
Effect of curcumin on the expressions of TGF-β1 in peritoneum of experimental PF model. TGF-β1-positive cells were occasionally found in the peritoneum of the control group (**a**), but were increased in the PF group (**b**). Curcumin at 10 mg/kg (**c**), 20 mg/kg (**d**) and 40 mg/kg (**e**) all markedly reduced the number of TGF-β1-positive cells. **f** Quantification analysis of number of TGF-β1-positive cells in the peritoneum (cells/mm^2^) in each group. **g** Quantitative real-time PCR showed the TGF-β1 mRNA expression in the peritoneum of each group. ANOVA was performed and followed with Tukey’s post hoc test. ***P < 0.001 vs. control group; ##*P* < 0.01, ###*P* < 0.001 vs. PF group

TGF-β1 plays a major role in peritoneal EMT and fibrosis. To study the mechanisms of curcumin action, the expression of TGF-β1 was determined by qPCR, ELISA and immunohistochemical staining. Compared with the control rats, the contents of TGF-β1 proteins were increased in peritoneal fluid of PD rats, and was significantly decreased by curcumin administration for 8 weeks (*P* < 0.05) (Fig. 2c). Immunohistochemistry showed that few TGF-β1-positive cells in peritoneum was detected in control rats (Fig. 3a), which was increased in PD rats (Fig. 3b). However, Curcumin markedly decreased TGF-β1-positive cells at all three doses expression of TGF-β1 (Fig. 3c, d, e). Quantitative analysis showed that the number of TGF-β1-positive cells in the peritoneum was significanfly reduced by curcumin (Fig. 3f). In parallel, TGF-β1 mRNA levels were increased in the PD group, but significantly reduced in the PD + curcumin (20 and 40 mg/kg) group (Fig. 3g).

### Effect of Curcumin on α-SMA and collagen I expression PD rats

**Fig. 4:**
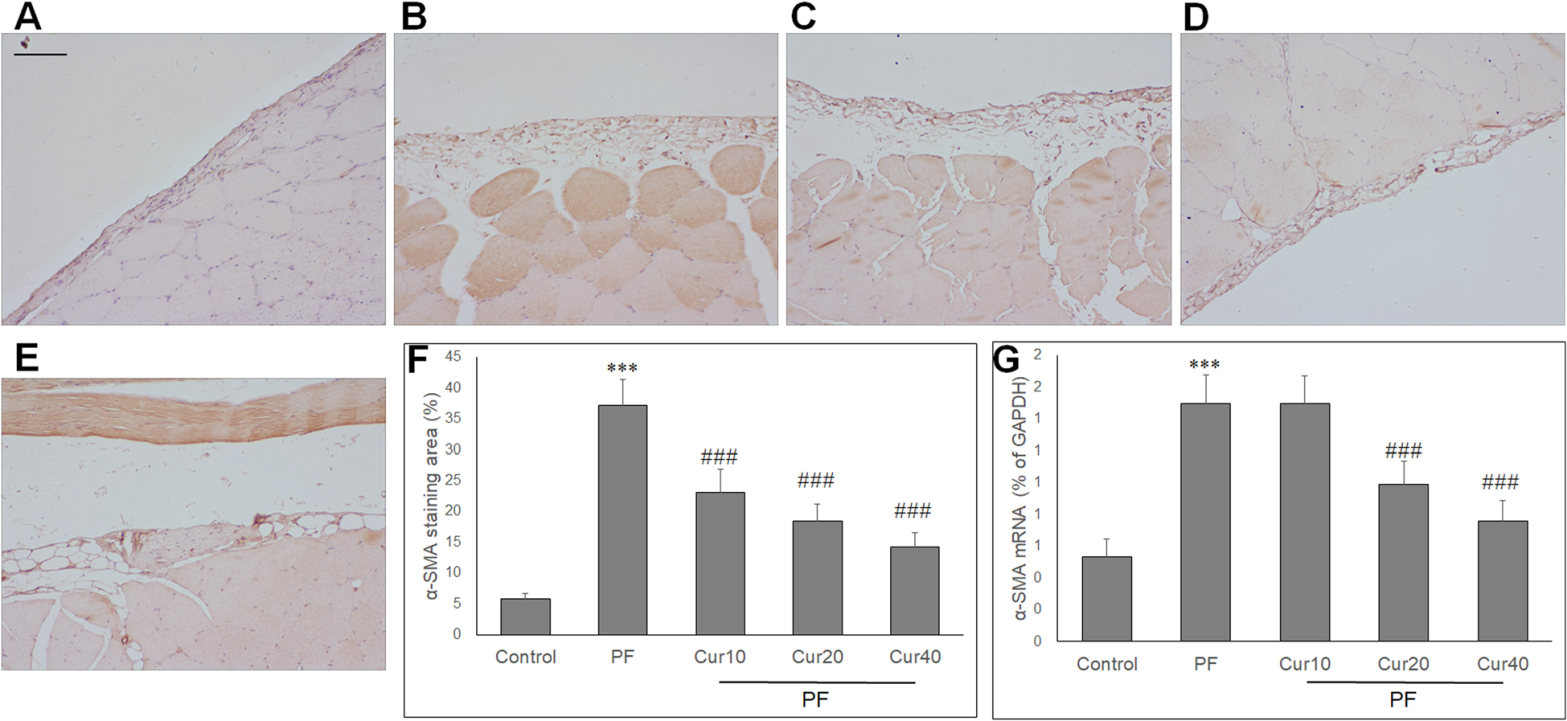
Effect of curcumin on the expressions of α-SMA in peritoneum of experimental PF model. Peritoneal expression of α-SMA (myofibroblasts) was determined by immunohistochemistry, and presented as α-SMA staining area (%). **a** No α-SMA expression is found in the peritoneum of the control group. **b** After induction of PF, high α-SMA expression is detected. Curcumin showed markedly reduced expression of α-SMA at 10 mg/kg (**c**), 20 mg/kg (**d**) and 40 mg/kg (**e**) compared with the PF group. **f** The α-SMA expression in the peritoneum presented as α-SMA staining area (%) in each group. **g** qRT PCR showed the mRNA expression in the peritoneum of each group. ANOVA was performed and followed with Tukey’s post hoc test. ***P < 0.001 vs. control group; ##*P* < 0.01, ###*P* < 0.001 vs. PF group

**Fig. 5:**
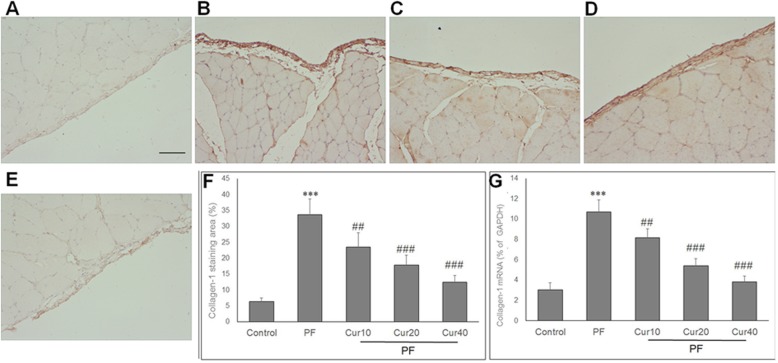
Effect of curcumin on the expressions of collagen I in peritoneum of experimental PF model. Peritoneal expression of collagen I presented as staining area (%). Few collagen I were observed in the peritoneum of the control rats (**a**), but high collagen I expression were found in the PF rats (**b**). Curcumin at 10 mg/kg (**c**), 20 mg/kg (**d**) and 40 mg/kg (**e**) all markedly reduced the collagen I staining area. **f** Quantification analysis of the collagen I staining area in the peritoneum (%) in each group. **g** Quantitative real-time PCR showed the collagen I mRNA expression in the peritoneum of each group. ANOVA was performed and followed with Tukey’s post hoc test. ***P < 0.001 vs. control group; ##*P* < 0.01, ###*P* < 0.001 vs. PF group

To investigate the potential role of EMT in reduced peritoneal fibrosis by curcumin*,* we then measured α-SMA and collagen I expression in peritoneum. The expression of α-SMA (Fig. 4a) and collagen I (Fig. 5a) were scarce in the control group, while in PD rats, α-SMA (Fig. 4b) and collagen I (Fig. 5b) expressions in the peritoneum were increased. Treatment with curcumin significantly decreased the α-SMA (Fig. 4c, d, e) and collagen I (Fig. 5c, d, e) expression in the peritoneal membrane. Quantitative analysis showed that curcumin significantly reduced the α-SMA (Fig. 4f) and collagen I (Fig. 5f) staining area in the peritoneum of PD rats. In parallel, curcumin also significantly reduced α-SMA and collagen I mRNA levels at 20 and 40 mg/kg doses in the PD rats (Figs. 4g and 5g).

### Curcumin inhibited activation of TAK1 pathway

**Fig. 6:**
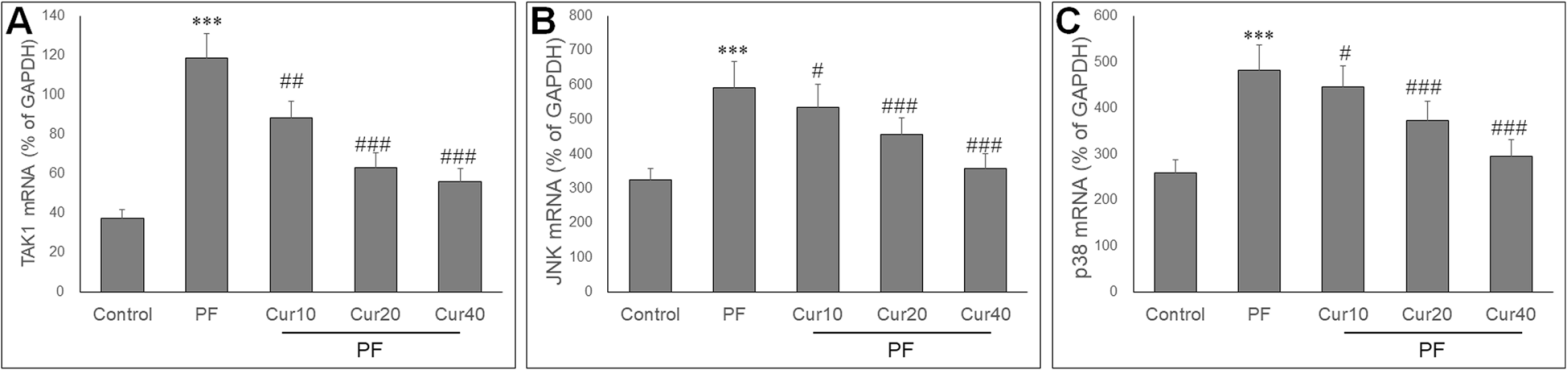
Effect of curcumin on mRNA expressions of TAK1, JNK and p38 in PF rats. qRT PCR showed that after induction of PF, the mRNA levels of TAK1 (**a**), JNK (**b**) and p38 (**c**) were significantly increased. Curcumin markedly reduced mRNA levels of TAK1, JNK and p38 at all doses in PF rats. ANOVA was performed and followed with Tukey’s post hoc test. ***P < 0.001 vs. control group; #*P* < 0.05, ###*P* < 0.001 vs. PF group

To investigate the molecular mechanisms underlying suppressed peritoneal fibrosis by curcumin, we measured expression of TAK1, JNK and p38. qRT-PCR showed that the mRNA expressions of TAK1, JNK and p38 were significantly up-regulated in PD rats (*P* < 0.05). Treatment with curcumin attenuated the up-regulation of TAK1, JNK and p38 in the peritoneum of PD rats (*P* < 0.05) (Fig. 6a, b, c).

**Fig. 7:**
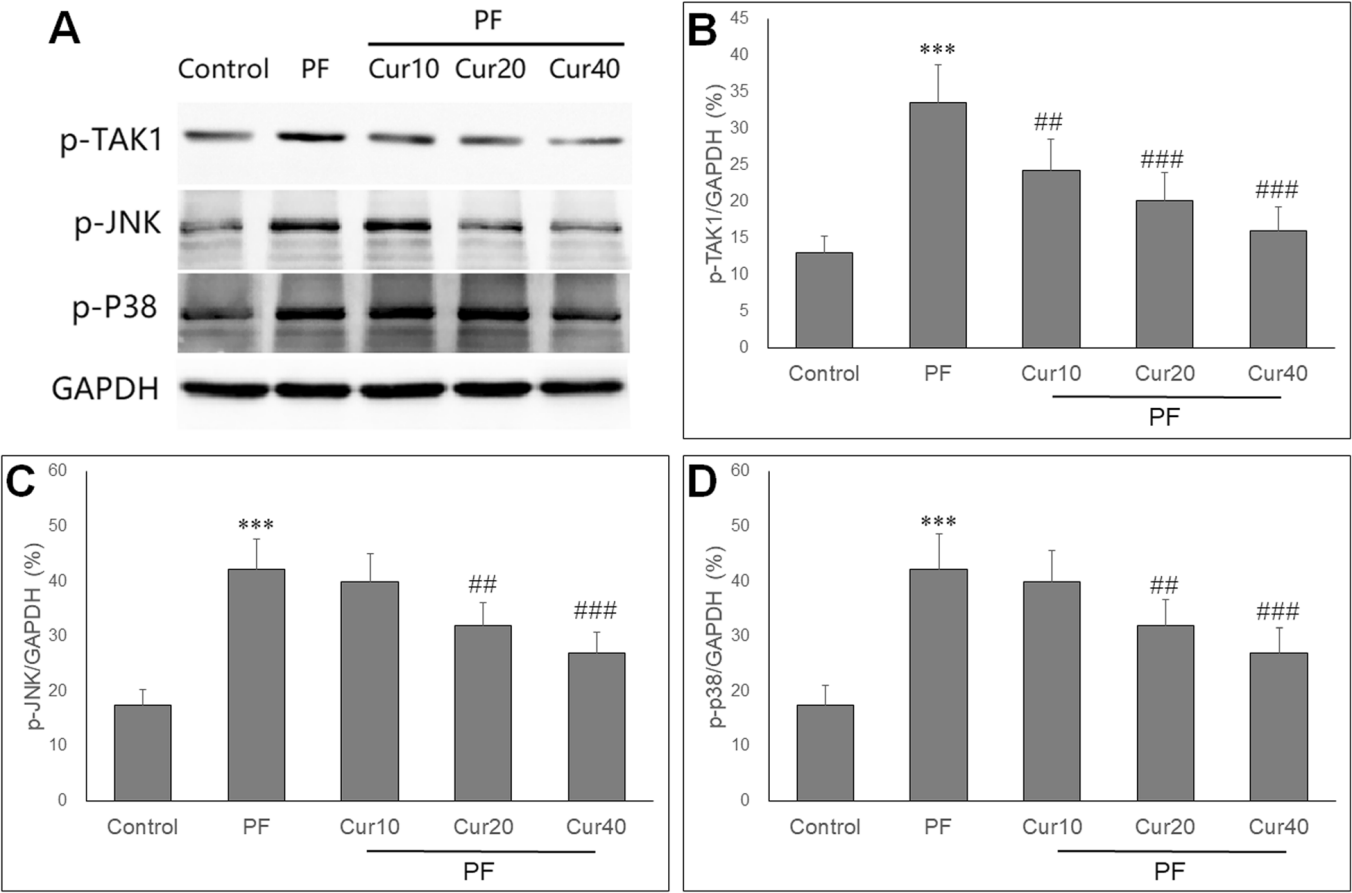
Effect of curcumin on protein expressions of TAK1, JNK and p38 in PF rats. **a** Representative immunoblots of p-TAK1, p-JNK, p-p38 and GAPDH in PF rats of each group were shown. After induction of PF, the protein levels of p-TAK1 (**b**), p-JNK (**c**) and p-p38 (**d**) were significantly increased. Curcumin markedly reduced protein levels of p-TAK1, p-JNK and p-p38 at moderate (20 mg/kg) and high (40 mg/kg) doses in PF rats. ANOVA was performed and followed with Tukey’s post hoc test. ***P < 0.001 vs. control group; ##*P* < 0.01, ###*P* < 0.001 vs. PF group

Western blot analysis showed the protein expressions of these three genes in peritoneum, as evidenced by representative images of decreased p-TAK1, p-JNK and p-p38 protein by curcumin treatment (Fig. 7a). Quantitative analysis showed that the protein expressions of p-TAK1, p-JNK and p-p38 were increased in PD rats, but were significantly decreased by curcumin treatment at 20 and 40 mg/kg doses (*P* < 0.05) (Fig. 7b, c, d).

## Discussion

Peritoneal fibrosis is defined as morphologic and functional changes to the peritoneal membrane (PM) caused by long-term peritoneal dialysis. Peritoneal fibrosis can lead to loss of peritoneal ultrafiltration capacity, with inflammation and angiogenesis as key events of pathogenesis [19]. Currently, no effective therapy for peritoneal fibrosis have been approved in clinic. Most studies thus far have focused on herbal medicine as an alternative treatment.

Curcumin, a plant polyphenol derived from the turmeric. As a traditional used herbal medicine and also a food spice in global cuisines, turmeric was reported to have extensively clinical applications in various kinds of diseases, such as asthma, fibrosis, diabetes, and abdominal pain [20]. A lot of emerging evidences have indicated that curcumin shows anti-fibrotic effects on organ fibrosis [10–13, 21]. However, the potential of curcumin to serve as therapeutic agents to ameliorate peritoneal membrane damage, as well as their mechanisms of action, have not yet been clearly delineated. In this study, we showed that curcumin treatment ameliorates the peritoneal damage induced by PD fluids in a PD rat model. The positive effects of curcumin include preservation of mesothelium, a reduction in fibrosis and an improvement of membrane function. In addition, our results suggest an implication of TAK1, p38 and JNK pathway in mediating the inhibitory effect of curcumin on PD-related PF.

High glucose, as a commonly used osmotic agent in most PD solutions, triggers changes in the morphology and function to the peritoneal membrane over time, which eventually causes peritoneum deterioration, and consequently leads to the occurrence of ultrafiltration failure (UFF) [22]. In our study, the results showed that when compared with the normal group, UFV decreased and MTG increased in the PD rats. However, the UFV elevated and MTG reduced were observed in rats which received curcumin (20 and 40 mg/kg) treatment groups. The results obtained indicated that curcumin treatment prevented UFF in PD rats.

Some studies have demonstrated that peritoneal fibrosis and the subsequent encapsulating peritoneal sclerosis (EPS) is always a result of long-term peritoneal dialysis [6]. In this study, in order to evaluate the inhibitory effects of curcumin on PD-related PF in vivo, we established a rat model of PF by short-term LPS stimulation and chronic infusing with 4.25% dextrose-monohydrate dialysis solution through a silicone catheter [23]. We choose this PF model because continuous exposure to nonphysiological PD solutions and episodes of peritonitis are presently the main cause of peritoneal dysfunction. After a 8-week model-building, several pathological alterations in the peritoneum were observed in the PD rats, including extensive interstitial fibrosis, mesothelial denudation, and changes in the peritoneum thickness, as previously described in the literature [24, 25]. However, the curcumin administration notably improved peritoneal function and relieved peritoneal fibrosis in a dose-dependent manner. For instance, the middle and high dose of curcumin significantly ameliorated the pathological alterations in the peritoneum, such as the interstitial fibrosis and the thickening of the peritoneum.

It has been identified that EMT of mesothelial cells (MCs) is an early and crucial process in the induction of peritoneal fibrosis during exposure to PD fluids [5, 6]. While epithelial features are lost, MCs rapidly gain expression of molecules related to EMT, such as α-SMA and fibroblast surface protein (FSP1). Moreover, MCs produce high levels of Plasminogen activator inhibitor-1(PAI-1), collagen I and fibronectin. The whole process can be induced by several growth factors, including TGF-β, which has been identified as a key mediator of mesothelial EMT both in vitro [26] and in vivo [27]. Increasingly evidence suggests that treatment to inhibit EMT could prevent peritoneal fibrosis and therefore preserve the peritoneal membrane [6, 28, 29]. In order to evaluate if the inhibitory effects of curcumin on expression of EMT markers in vivo, we specifically analyzed the expression of TGF-β, α-SMA and collagen I, which are the representative markers implicated in EMT. Our results showed that TGF-β, α-SMA and collagen I expression in mRNA and protein levels were significantly increased in the peritoneum of PD rats, and this changes were significantly attenuated by treatment with medium to large curcumin*.* Meanwhile, the concentrations of TGF-β1 in peritoneal fluid of PD rats were detected. The result showed that increased TGF-β1 levels in peritoneal fluid of PD rats were significantly down-regulated by curcumin administration for 7 weeks. This result is consistent with another report that TGF-β1 is present in fluids from patients undergoing PD and its levels correlate with deterioration of peritoneal membrane [19]. Taken together, it appears that the mechanism by which curcumin anti-fibrotic effects on peritoneal fibrosis is partly through inhibiting of TGF-β1-induced EMT of peritoneal mesothelial cells. Curcumin was proved to ameliorate TGF-β1-induced EMT in the suppression of renal fibrosis [30] and liver fibrosis [31].

The smad signaling pathway is widely accepted as a canonical pathway induced by TGF-β1 in the induction of EMT and its reversal. Smad2–3 are directly induced by TGF-β1 and have a primary role in peritoneal EMT and fibrosis. Besides this, the induction of EMT and fibrosis may be regulated by smad-independent pathways. TAK1, a serine/ threonine kinase, which has emerged as a critical signaling molecule in TGF-β1-induced Smad-independent signaling pathways. After activated by TGF-β1, TAK1 can activate JNK and p38, respectively, thus regulating the transcription of target genes [32]. Recently, TGF-β1/TAK1 pathway has been identified as an important participant process of TGF-β1-induced fibrosis. Inhibition of TAK1 suppressed EMT of primary human mesothelial cells [17] and inhibited peritoneal fibrosis of rats with long-term peritoneal dialysis [33]. To study the mechanisms of curcumin action, we tested the expression of TAK1, JNK and p38 in peritoneum by qPCR and Western blot. The results showed that TAK1, JNK and p38 mRNA and p-TAK1, p-JNK and p-p38 protein levels were significantly increased in the peritoneum of PD rats. Compared to the level in PD rats, TAK1, JNK and p38 expression in the peritoneum was reduced following treatment with a middle and high dose of Curcumin. Therefore, our study shows that curcumin suppresses peritoneal fibrosis partly through inhibiting TGF-β1/TAK1 pathway, which is different from reduced renal fibrosis by curcumin through PPAR-γ and Smad pathways downstream of TGF-β1 [34, 35].

In conclusion, It has been demonstrated that administration of curcumin to rats exposed to PD fluid produce pleiotropic protective effects on the peritoneal membrane, reducing inflammation and TGF-β1expression, as well as preserving mesothelial cell monolayer. In addition to the histological findings, curcumin improves peritoneal functions. Our results demonstrate that curcumin showed an obvious protective effect on PD-related PF and suggest an implication of TAK1, p38 and JNK pathway in mediating the benefical effects of curcumin. These findings indicate potential therapeutic effect of curcumin on peritoneal fibrosis. However, future studies are needed to elucidate detailed mechanisms including PPAR-γ and Smad pathways downstream of TGF-β1, and to translate this finding into clinical application.

## Conclusions

Present results demonstrate that curcumin showed a protective effect on PD-related PF and suggest an implication of TAK1, p38 and JNK pathway in mediating the benefical effects. These findings suggest that curcumin may be a potential agent for the prevention and treatment of peritoneal fibrosis.

## Data Availability

All data generated or analyzed during this study are included in this published article and its supplementary information files.

## Abbreviations

EMT: Epithelial–mesenchymal transition
EPS: Encapsulating peritoneal sclerosis
ESRD: End-stage renal disease
FSP1: Fibroblast surface protein
GDPs: Glucose degradation products
JNK: c-Jun N-terminal kinase
MCs: Mesothelial cells
MTG: Mass transfer of glucose
PAI-1: Plasminogen activator inhibitor-1
PD: Peritoneal dialysis
PET: Peritoneal Equilibration Test
PF: Peritoneal fibrosis
PM: Peritoneal membrane
PMCs: Peritoneal mesothelial cells
TAK1: Transforming growth factor-activated kinase-1
TGF-β1: Transforming growth factor-β1
UFF: Ultrafiltration failure
UFV: Ultrafiltration volume
α-SMA: α smooth muscle actin
